# The Hydrangea Arborescens

**Published:** 1885-07

**Authors:** 


### THE HYDRANGEA ARBORESCENS.

The value of this native plant in renal affections was first made known to the medical public by the former editor of this journal, Dr. S. W. Butler. Recently, Lambert & Co., of St. Louis, have combined the active elements of the plant with lithia i. a preparation called "Lithiated Hydrangea," which unites the virtue of both these two remedies. In the *Chicago Weekly Review*, two cases of rheumatic gout with renal complications are reported by Dr. F. S. Senier, of Waukesha, Wis., where this preparation in doses of a drachm, thrice daily, largely diluted, acted with prompt and satisfactory effect. The combination seems to us a happy one.—*Medical and Surgical Reporter, Philadelphia.*

